# Activation of the Nrf2/ARE signaling pathway ameliorates hyperlipidemia-induced renal tubular epithelial cell injury by inhibiting mtROS-mediated NLRP3 inflammasome activation

**DOI:** 10.3389/fimmu.2024.1342350

**Published:** 2024-04-24

**Authors:** Xu-shun Jiang, Ting Liu, Yun-feng Xia, Hua Gan, Wei Ren, Xiao-gang Du

**Affiliations:** ^1^ Department of Nephrology, The First Affiliated Hospital of Chongqing Medical University, Chongqing, China; ^2^ Department of Cardiology, The First Affiliated Hospital of Chongqing Medical University, Chongqing, China; ^3^ Department of Endocrinology, The First Affiliated Hospital of Chongqing Medical University, Chongqing, China; ^4^ The Chongqing Key Laboratory of Translational Medicine in Major Metabolic Diseases, The First Affiliated Hospital of Chongqing Medical University, Chongqing, China

**Keywords:** Nrf2, mitochondrial ROS, NLRP3 inflammasome, hyperlipidemia, renal tubular epithelial cells

## Abstract

Dyslipidemia is the most prevalent independent risk factor for patients with chronic kidney disease (CKD). Lipid-induced NLRP3 inflammasome activation in kidney-resident cells exacerbates renal injury by causing sterile inflammation. Nuclear factor erythroid 2-related factor 2 (Nrf2) is a transcription factor that modulates the cellular redox balance; however, the exact role of Nrf2 signaling and its regulation of the NLRP3 inflammasome in hyperlipidemia-induced kidney injury are poorly understood. In this study, we demonstrated that activation of the mtROS–NLRP3 inflammasome pathway is a critical contributor to renal tubular epithelial cell (RTEC) apoptosis under hyperlipidemia. In addition, the Nrf2/ARE signaling pathway is activated in renal tubular epithelial cells under hyperlipidemia conditions both *in vivo* and *in vitro*, and Nrf2 silencing accelerated palmitic acid (PA)-induced mtROS production, mitochondrial injury, and NLRP3 inflammasome activation. However, the activation of Nrf2 with tBHQ ameliorated mtROS production, mitochondrial injury, NLRP3 inflammasome activation, and cell apoptosis in PA-induced HK-2 cells and in the kidneys of HFD-induced obese rats. Furthermore, mechanistic studies showed that the potential mechanism of Nrf2-induced NLRP3 inflammasome inhibition involved reducing mtROS generation. Taken together, our results demonstrate that the Nrf2/ARE signaling pathway attenuates hyperlipidemia-induced renal injury through its antioxidative and anti-inflammatory effects through the downregulation of mtROS-mediated NLRP3 inflammasome activation.

## 1 Introduction

Dyslipidemia is a common metabolic disorder in patients with chronic kidney disease (CKD), including diabetic kidney disease (DKD), and is an independent risk factor for the progression of CKD (1). It is now generally accepted that excess lipid accumulation in the kidney critically contributes to the pathogenesis of diabetic nephropathy (DN) and is closely associated with a progressive decline in renal function (2, 3). In proteinuric kidney disease, including DN, FFA-bound albumin is filtered through the injured glomeruli and reabsorbed by the proximal tubule, which may promote the progression of renal tubular cell damage and tubulointerstitial fibrosis (4, 5).

The NOD-like receptor protein 3 (NLRP3) inflammasome is a cytosolic multiprotein complex that contains NLRP3, apoptosis-related speck protein (ASC), and cysteine aspartate-1 precursor (pro-caspase-1). Moreover, the NLRP3 assembly activates caspase-1, thereby mediating the maturation and secretion of the proinflammatory cytokines IL-1β and IL-18. Previous studies have implicated the activated NLRP3 inflammasome in the pathogenesis of many kidney diseases, including acute kidney injury (AKI), crystal-related nephropathy, and CKD (6). In addition to the key role of the NLRP3 inflammasome in immune cells, previous studies have also demonstrated inflammasome activation in nonimmune cells in the kidney, such as podocytes (7) and renal tubular epithelial cells (8), in kidney disease. Activation of the NLRP3 inflammasome in kidney-resident cells drives sterile inflammation and promotes kidney dysfunction in the context of CKD. It has been reported that the NLRP3 inflammasome is activated in response to sterile stimuli such as hyperglycemia (8), hyperlipidemia (9), angiotensin II (10), and aldosterone (11) in renal tubular epithelial cells. However, the exact mechanism by which hyperlipidemic conditions induce NLRP3 inflammasome activation in renal tubular epithelial cells still needs to be clarified.

Nuclear factor erythroid 2-related factor 2 (Nrf2) is a master regulator that modulates the cell redox balance in response to oxidative stress. Under normal conditions, Nrf2 localizes to the cytoplasm by interacting with Kelch ECH-associated protein 1 (Keap1), a physiological inhibitor of Nrf2 that facilitates Nrf2 ubiquitination and degradation. Under oxidative stress conditions, Nrf2 is released from Keap1, translocates into the nucleus, binds to antioxidant response elements (AREs), and promotes the transcription of antioxidant genes, including NADPH quinine oxidoreductase 1 (NQO1) and heme oxygenase-1 (HO-1) (12). Previous studies have verified that the Nrf2/ARE pathway plays a protective role in diverse human diseases, including inflammatory diseases (13), Parkinson’s disease (14), cardiovascular diseases (15), atherosclerosis (16), AKI, and CKD (17). Liu et al. reported that knocking out Nrf2 enhanced susceptibility to ischemic AKI and caused significantly worse renal function, tissue damage, vascular permeability, and mortality in Nrf2 knockout mice following renal I/R than in wild-type mice (18). Alaofi et al. showed that activation of the Nrf2 pathway ameliorated the progression of streptozotocin (STZ)-induced DN in rats (19). However, the potential role that Nrf2/ARE signaling pathways play in the pathogenesis of CKD with dyslipidemia has still not been fully elucidated.

In this study, we aimed to explore the role of the Nrf2/ARE signaling pathway and its regulation of mitochondrial ROS production and NLRP3 inflammasome activation in hyperlipidemia-induced renal tubular epithelial cell injury.

## 2 Materials and methods

### 2.1 Cell culture and treatment

Human proximal tubular cells (HK-2 cells) were cultured in DMEM/F-12 medium supplemented with 10% fetal bovine serum (FBS) in a humidified incubator containing 5% CO_2_ at 37°C. Transient transfection of HK-2 cells with small interfering RNA (siRNA) was conducted with Lipofectamine 2000 (Invitrogen, CA, USA), and siRNAs (Nrf2 siRNA F: 5′-GCCCAUUGAUGUUUCUGAUTT-3′, R: 5′-AUCAGAAACGAAAUCAAUGGGCTT-3′; scrambled siRNA F: 5′-UUCUCCGAACGUGUCACGUTT-3′, R: 5′-ACGUGACACGUUCGGAGAATT-3′) were synthesized by GenePharma Company (Shanghai, China). For the overexpression of Nrf2, cells were transfected with Nrf2-pcDNA3.1-3xflag-C plasmids (Fenghbio, Hunan, China) using Lipofectamine 2000 reagent. MCC950 (10 μM), Mito Tempol (100 μM), or tBHQ (30 μM) was added to the culture medium for 2 h before exposure to palmitic acid (PA) media.

### 2.2 Animals

Male Sprague−Dawley rats (*n* = 24, aged 4 weeks) were obtained from the Animal Center of Chongqing Medical University and housed under pathogen-free conditions with free access to food and water. The rats were divided randomly into the following three groups: a control group, a high-fat diet (HFD) group, and a HFD group treated with tBHQ. The control group rats were fed a normal chow diet (#D12450B; 10 kcal% fat; Research Diet), and the HFD group was fed a Western high-fat diet (#D12451; 45 kcal% fat; Research Diet). The HFD-fed rats in the treatment group were intraperitoneally injected with tBHQ (50 mg/kg, every other day for 12 weeks; Sigma−Aldrich, USA), whereas the control group rats were injected with an equal volume of normal saline. All groups of rats were sacrificed at 20 weeks of age, and blood and kidney tissue samples were obtained for further experimental analysis. All animal experiments were performed in strict accordance with the Ethics Committee of Chongqing Medical University.

### 2.3 Patients and tissue samples

Kidney tissue samples were collected from the Pathology Department of the First Affiliated Hospital of Chongqing Medical University. For the control group, kidney tissues were collected from six patients who underwent nephrectomy for renal trauma with no other evidence of renal disease. Kidney tissues were collected from eight CKD patients with hyperlipidemia who underwent renal biopsy. Informed consent was obtained from all patients, and the experiments were approved by the Ethics Committee of The First Affiliated Hospital of Chongqing Medical University. Nrf2 and NLRP3 expression was measured by immunofluorescence and immunohistochemistry.

### 2.4 Measurement of mitochondrial reactive oxygen species production and the mitochondrial membrane potential (ΔΨm)

To measure mitochondrial ROS, HK-2 cells were seeded on slides in 12-well culture plates. After treatment, live HK-2 cells were stained with 10 μM DCFH-DA and 50 nM MitoTracker Red for 30 min at 37°C or incubated with MitoSOX™ Red fluorescent probe (5 μM, M36008, Invitrogen, USA) at 37°C for 20 min. Moreover, 4-μm-thick frozen sections were stained with 5 μM MitoSOX™ Red fluorescent probe for 20 min at 37°C in the dark. After being washed three times with phosphate-buffered saline (PBS), the stained HK-2 cells and frozen kidney sections were immediately visualized under a fluorescence microscope. The mitochondrial membrane potential was evaluated by using JC-1 dye (Beyotime, China), and live HK-2 cells or frozen kidney sections were incubated with JC-1 staining solution for 20 min at 37°C in the dark. After the cells were washed three times with PBS, images were subsequently acquired with a fluorescence microscope.

### 2.5 Immunohistochemical staining

Paraffin-embedded renal tissue samples were sliced into 4-mm-thick sections. After deparaffinization in xylene and rehydration in a series of graded alcohols, the target antigens of the tissues were retrieved by microwaving for 15 min in 10 mmol/L sodium citrate buffer (pH 6.0), and endogenous peroxidases were inactivated by 0.3% H_2_O_2_ for 15 min at room temperature. After being washed, the sections were blocked in normal goat serum buffer for 15 min and then incubated with the following primary antibodies at 4°C overnight: rabbit anti-Nrf2 (1:200, Abcam), rabbit anti-HO-1 (1:200, Abcam), rabbit anti-NQO-1 (1:200, Abcam), rabbit anti-NLRP3 (1:200, CST), rabbit anti-caspase1 (1:200, CST), rabbit anti-IL-18 (1:200, Abcam), and mouse anti-8-OHdG (1:200, Santa Cruz). The sections were washed with PBS, incubated with a biotinylated secondary antibody (Zhongshan Golden Bridge Inc., China) for 30 min, incubated using a 3,3′-diaminobenzidine (DAB) kit for 3–5 min, counterstained with hematoxylin, dehydrated in ethanol, and visualized using a Nikon Eclipse 80i microscope. Quantification of the stained distribution area (area) and the integrated optical density (IOD) of the target protein in each IHC image was performed using ImageJ software. The average optical density (AOD = IOD/area) was used in this study for statistical analysis.

### 2.6 Immunofluorescence staining

HK-2 cells were seeded on slides in 12-well culture plates. After various treatments, the cells were stained with MitoTracker Red at 37°C for 30 min in the dark, fixed with 4% paraformaldehyde for 10 min, and permeabilized in 0.1% Triton X-100 for 15 min at room temperature. After blocking with 5% BSA buffer, the cells were subsequently incubated with the indicated primary antibodies against NLRP3 (1:200), 8-OHdG (1:200), or Nrf2 (1:200) at 4°C overnight. After washing three times with PBS, the cells were incubated with the corresponding FITC-conjugated secondary antibodies and stained with DAPI. The images were captured using a fluorescence microscope.

### 2.7 Western blot analysis

Proteins from kidney tissues or HK-2 cells were electrophoretically separated on 8%–10% SDS−polyacrylamide gels and then transferred to PVDF membranes (Millipore, USA). The membranes were blocked with 5% nonfat milk and incubated overnight at 4°C with the following primary antibodies: rabbit anti-NLRP3 (1:1,000, CST), rabbit anti-caspase1 (1:1,000, CST), rabbit anti-IL-18 (1:1,000, Abcam), rabbit anti-Nrf2 (1:1,000, Abcam), rabbit anti-HO-1 (1:5,000, Abcam), rabbit anti-NQO-1 (1:5,000, Abcam), rabbit anti-cleaved-caspase3 (1:1,000, CST), and mouse anti-β-actin (1:5,000, Sungene Biotech). Finally, the membranes were washed three times with TBST and then incubated with the corresponding horseradish peroxidase (HRP)-conjugated secondary antibodies for 1 h, after which the protein bands were visualized using an electrochemiluminescence detection system (GE Healthcare, Piscataway, NJ, USA).

### 2.8 Analysis of cell apoptosis

Cell apoptosis was detected using an Annexin V-FITC/PI apoptosis assay kit (Sungene Biotech, China). In brief, after double-staining with annexin V and propidine iodide (PI), the number of apoptotic cells was measured by flow cytometry analysis. To assess the extent of renal apoptosis, terminal deoxynucleotidyl transferase dUTP nick end-labeling (TUNEL) staining (*In Situ* Cell Death Detection Kit, POD, Roche) was performed on frozen kidney sections, and the number of TUNEL-positive cells per 0.25 mm^2^ was calculated.

### 2.9 Statistical analysis

All data are expressed as means ± SEM. Qualitative data were analyzed using Student’s *t*-test for comparisons between two groups or one-way analysis of variance (ANOVA) followed by Tukey’s posttest for comparisons among multiple groups. A value of *P <*0.05 was considered to indicate statistical significance.

## 3 Results

### 3.1 Activation of the NLRP3 inflammasome mediated high-lipid-induced tubular epithelial cell apoptosis

**Figure 1:**
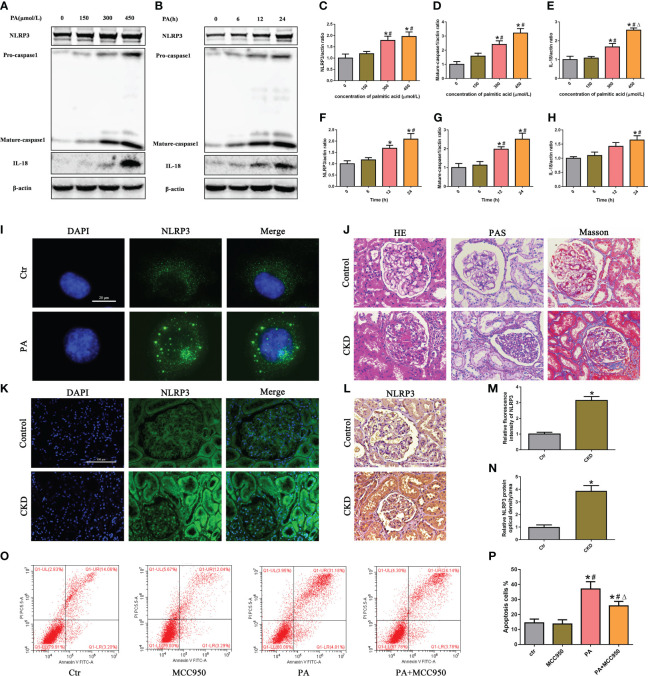
Activation of the NLRP3 inflammasome mediated high-lipid-induced tubular epithelial cell apoptosis. **(A, B)** Western blot analyses of the protein expression of NLRP3, caspase-1, and IL-18 in HK-2 cells after treatment with different concentrations of palmitic acid (PA) for 24 h or treatment with 300 μmol/L PA for different durations. **(C–H)** Quantification of NLRP3, caspase-1, and IL-18 expression in **(A, B)** (*n* = 3; *P < 0.05 vs. 0 μmol/L or 0 h, ^#^ *P* < 0.05 vs. 150 μmol/L or 6 h, and ^Δ^ *P* < 0.05 vs. 300 μmol/L). **(I)** Representative images of immunofluorescence staining for NLRP3 in HK-2 cells. **(J)** Representative images of kidney sections subjected to H&E, PAS, and Masson staining. **(K)** Representative image of immunofluorescence staining for NLRP3 in kidney sections. **(L)** Representative images of immunohistochemical staining for NLRP3 in kidney sections. **(M)** Quantification analysis of the fluorescence intensities of NLRP3 **(K)** (*n* = 6, *P < 0.05 vs. control group). **(N)** Quantification analysis of IHC staining for NLRP3 in (**L**) (*n* = 6, *P < 0.05 vs. control group). **(O)** Flow cytometry analysis of cell apoptosis in HK-2 cells. **(P)** Quantification analysis of apoptotic cells in **(O)** (*n* = 3; *P < 0.05 vs. control group, ^#^ *P* < 0.05 vs. MCC950 group, and ^Δ^ *P* < 0.05 vs. PA group).

To evaluate the activation of the NLRP3 inflammasome in PA-induced tubular epithelial cells, we determined the expression levels of NLRP3, mature caspase-1, and the proinflammatory cytokine IL-18, which are used as markers of inflammasome activation. As shown in **Figures 1A–H**, the protein expression of NLRP3, mature caspase-1, and IL-18 was significantly upregulated in HK-2 cells following PA stimulation in a dose- and time-dependent manner, as determined by western blot analyses. Moreover, immunofluorescence analysis revealed that PA induced an increase in NLRP3 protein expression compared to that in control cells (**Figure 1I**).

Next, we detected the expression of NLRP3 in the kidney tissue of CKD patients with hyperlipidemia. First, H&E and PAS staining revealed marked glomerular mesangial expansion and increased mesangial matrix in the kidneys of CKD patients with hyperlipidemia (**Figure 1J**). Masson’s trichrome staining revealed that renal fibrosis was enhanced in the kidneys of CKD patients with hyperlipidemia (**Figure 1J**). Subsequently, immunofluorescence analysis and immunohistochemical staining revealed that the expression of NLRP3 was significantly upregulated in the kidneys of CKD patients with hyperlipidemia (**Figures 1K–N**). Finally, we investigated the role of NLRP3 inflammasome activation in PA-induced HK-2 cell apoptosis. Flow cytometry showed that MCC950 (a NLRP3 antagonist) treatment significantly ameliorated PA-induced cell apoptosis (**Figures 1O, P**). Taken together, these results indicated that high lipid levels induced NLRP3 inflammasome activation, which promoted tubular epithelial cell apoptosis.

### 3.2 Role of mtROS in PA-induced mitochondrial damage and NLRP3 inflammasome activation in HK-2 cells

**Figure 2:**
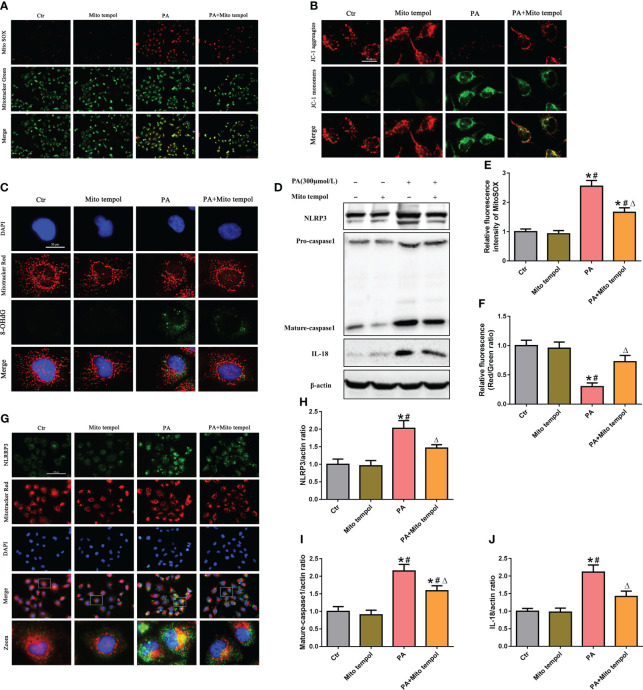
Role of mtROS in PA-induced mitochondrial damage and NLRP3 inflammasome activation in HK-2 cells. **(A)** Representative fluorescence images of MitoSOX and MitoTracker Green staining in different groups. **(B)** Representative fluorescence images of JC-1 staining in different groups. **(C)** Representative images of immunofluorescence double-labeled 8-OHdG and mitochondria (MitoTracker Red) in different groups. **(D)** Western blot analyses of the protein expression of NLRP3, caspase-1, and IL-18 in HK-2 cells. **(E, F)** Quantification of the fluorescence intensities of MitoSOX and JC-1 in **(A)** and **(B)** (*n* = 3; *P < 0.05 vs. control group, ^#^ *P* < 0.05 vs. Mito Tempol group, and ^Δ^ *P* < 0.05 vs. PA group). **(G)** Representative images of immunofluorescence double-labeled NLRP3 and mitochondria (MitoTracker Red) in the different groups. **(H–J)** Quantification of NLRP3, caspase-1, and IL-18 expression in **(D)** (*n* = 3; *P < 0.05 vs. control group, ^#^ *P* < 0.05 vs. Mito Tempol group, and ^Δ^ *P* < 0.05 vs. PA group).

Mitochondria are major sources of ROS and are also targets of ROS. To investigate the role of mtROS in mitochondrial damage, HK-2 cells were treated with the mitochondrion-targeted antioxidant Mito Tempol. Immunofluorescence analysis revealed that Mito Tempol treatment significantly attenuated the production of mtROS (**Figures 2A, E**) and reversed the loss of (the mitochondrial membrane potential) ΔΨm in HK-2 cells under PA stimulation (**Figures 2B, F**). Nuclear and mitochondrial DNA oxidative damage can be assessed by immunofluorescence staining for nuclear and nonnuclear 8-OHdG, respectively (20). As shown in **Figure 2C**, immunofluorescence analysis demonstrated that PA induced an increase in the colocalization of 8-OHdG and mitochondria or nuclei, suggesting that PA induced mitochondrial and nuclear DNA oxidative damage in HK-2 cells. However, Mito Tempol treatment significantly ameliorated PA-induced oxidative damage to mitochondrial and nuclear DNA in HK-2 cells.

Mitochondrial damage plays a pivotal role in NLRP3 inflammasome activation, and previous studies revealed that mtROS generated by damaged mitochondria are critically involved in NLRP3 inflammasome activation (21). Similarly, our Western blot analysis showed that Mito Tempol treatment significantly prevented the PA-induced increase in the expression of NLRP3, caspase-1, and IL-18 (**Figures 2D, H–J**). As mitochondria can serve as a molecular platform for the assembly of NLRP3 inflammasome components (22, 23), which subsequently mediate the maturation and secretion of proinflammatory cytokines, we explored the subcellular localization of NLRP3 inflammasome components under PA stimulation conditions and found that NLRP3 was located in the cytoplasm but not within mitochondria in control cells. However, stimulation with PA led to the recruitment of NLRP3 within mitochondria, as demonstrated by the increasing overlap of NLRP3 in the mitochondria (**Figure 2G**). However, Mito Tempol treatment inhibited PA-induced NLRP3 upregulation and recruitment within mitochondria (**Figure 2G**). These results suggested that PA-induced NLRP3 inflammasome activation was dependent on mitochondrial ROS generation.

### 3.3 The Nrf2/ARE signaling pathway was activated in high-lipid-induced tubular epithelial cells

**Figure 3:**
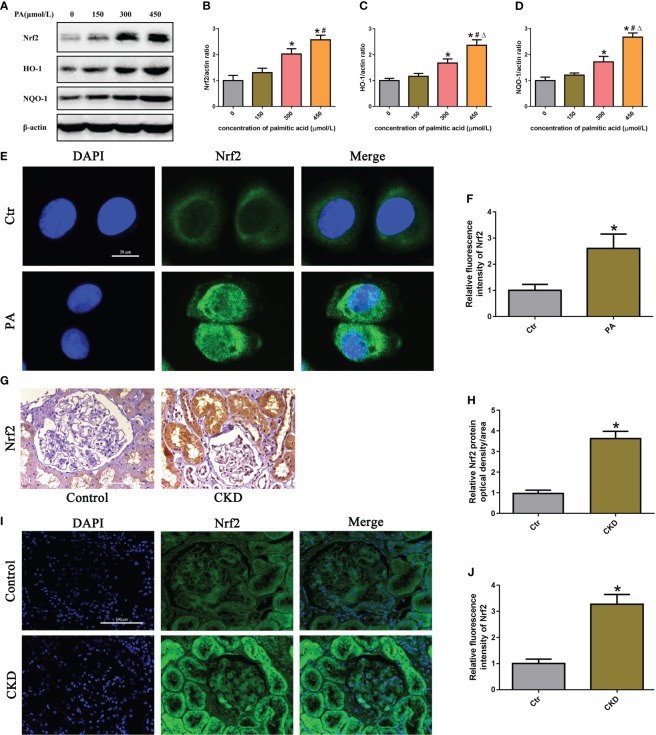
The Nrf2/ARE signaling pathway was activated in high-lipid-induced tubular epithelial cells. **(A)** Western blot analyses of the protein expression of Nrf2, HO-1, and NQO-1 in HK-2 cells. **(B–D)** Quantification of Nrf2, HO-1, and NQO-1 expression in **(A)** (*n* = 3; *P < 0.05 vs. 0 μmol/L, ^#^ *P* < 0.05 vs. 150 μmol/L, and ^Δ^ *P* < 0.05 vs. 300 μmol/L). **(E)** Representative images of immunofluorescence staining for Nrf2 in HK-2 cells. **(F)** Quantification analysis of the fluorescence intensities of Nrf2 in **(E)** (*n* = 3, *P < 0.05 vs. control group). **(G)** Representative images of immunohistochemical staining for Nrf2 in kidney sections. **(H)** Quantification analysis of IHC staining for Nrf2 in **(G)** (*n* = 6, *P < 0.05 vs. control group). **(I)** Representative images of immunofluorescence staining for Nrf2 in kidney sections. **(J)** Quantification analysis of the fluorescence intensities of Nrf2 in **(I)** (*n* = 6, *P < 0.05 vs. control group).

To determine the effects of PA exposure on the Nrf2/ARE signaling pathway, we measured the expression of Nrf2, HO-1, and NQQ-1 in HK-2 cells and found that they were noticeably increased in a dose-dependent manner under PA stimulation (**Figure 3A–D**). Additionally, we observed the expression and location of Nrf2 in HK-2 cells. Immunostaining revealed that PA treatment increased the Nrf2 fluorescence intensity and increased the colocalization of Nrf2 with the nucleus, indicating that Nrf2 translocated from the cytoplasm to the nucleus (**Figures 3E, F**). Furthermore, we detected the expression of Nrf2 in the kidney tissue of CKD patients with hyperlipidemia. As shown in **Figures 3G–J**, immunohistochemical staining and immunofluorescence analysis revealed that the expression of Nrf2 was significantly upregulated in the kidneys of CKD patients with hyperlipidemia. These results demonstrated that high lipid concentrations activate the Nrf2/ARE signaling pathway in tubular epithelial cells.

### 3.4 Silencing of Nrf2 enhanced mtROS production and mitochondrial damage in PA-treated HK-2 cells

**Figure 4:**
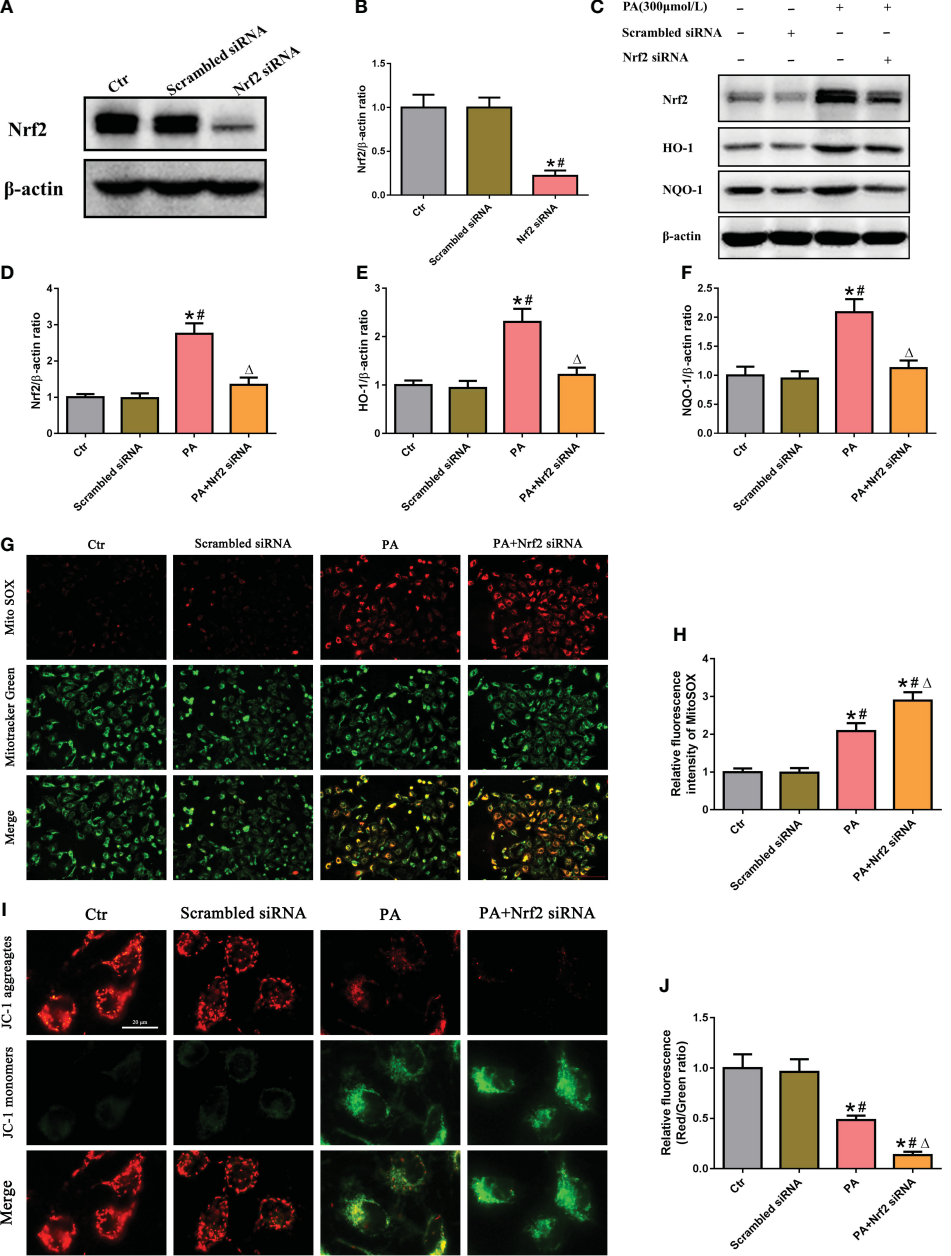
Silencing of Nrf2 enhanced mtROS production and mitochondrial damage in PA-treated HK-2 cells. **(A)** Western blot analyses of the protein expression of Nrf2 in HK-2 cells after transfection with scrambled siRNA or Nrf2 siRNA. **(B)** Quantification analysis of Nrf2 expression in **(A)** (*n* = 3; *P < 0.05 vs. control group and ^#^ *P* < 0.05 vs. scrambled siRNA group). **(C)** Western blot analyses of the protein expression of Nrf2, HO-1, and NQO-1 in different groups. **(D–F)** Quantification analysis of Nrf2, HO-1, and NQO-1 expression in **(C)** (*n* = 3; *P < 0.05 vs. control group, ^#^ *P* < 0.05 vs. scrambled siRNA group, and ^Δ^ *P* < 0.05 vs. PA group). **(G)** Representative fluorescence images of MitoSOX and MitoTracker Green staining in different groups. **(H)** Quantification of the fluorescence intensities of MitoSOX in **(G)** (*n* = 3; *P < 0.05 vs. control group, ^#^ *P* < 0.05 vs. scrambled siRNA group, and ^Δ^ *P* < 0.05 vs. PA group). **(I)** Representative fluorescence images of JC-1 staining in different groups. **(J)** Quantification of the fluorescence intensities of JC-1 in **(I)** (*n* = 3; *P < 0.05 vs. control group, ^#^ *P* < 0.05 vs. scrambled siRNA group, and ^Δ^ *P* < 0.05 vs. PA group).

To determine the role of the Nrf2/ARE signaling pathway in mtROS production and mitochondrial function in PA-treated HK-2 cells, siRNA was used to silence Nrf2. Immunoblot analysis indicated that Nrf2 siRNA successfully suppressed the protein expression of Nrf2 (**Figures 4A, B**), and Nrf2 silencing significantly decreased the protein expression of Nrf2, HO-1, and NQO-1 in HK-2 cells treated with or without PA (**Figures 4C–F**). In addition, immunofluorescence staining showed that silencing Nrf2 significantly exacerbated mtROS production (**Figures 4G, H**) and the loss of ΔΨm (**Figures 4I, J**) in HK-2 cells cultured in PA media. These data suggested that inhibiting the Nrf2/ARE signaling pathway exacerbated PA-induced mtROS production and mitochondrial damage in HK-2 cells.

### 3.5 Activation of the Nrf2/ARE signaling pathway ameliorated PA-induced mtROS production and mitochondrial damage in HK-2 cells

**Figure 5:**
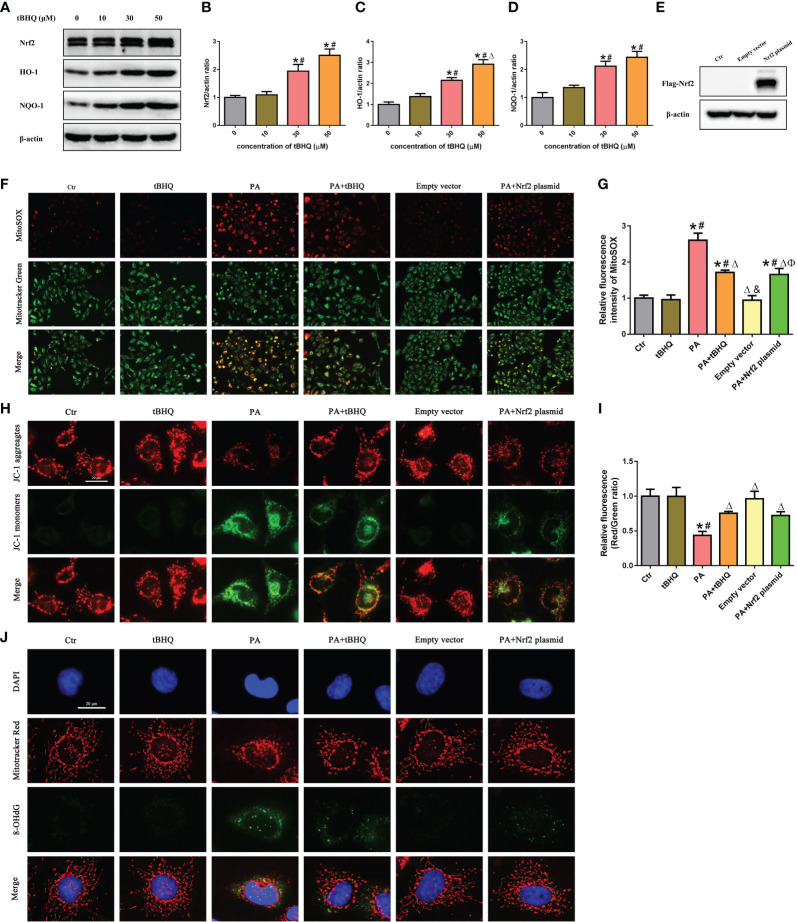
Activation of the Nrf2/ARE signaling pathway ameliorated PA-induced mtROS production and mitochondrial damage in HK-2 cells. **(A)** Western blot analyses of the protein expression of Nrf2, HO-1, and NQO-1 in HK-2 cells after being treated with various concentrations of tBHQ (0, 10, 30, and 50 μM). **(B–D)** Quantification analysis of Nrf2, HO-1, and NQO-1 expression in **(A)** (*n* = 3; *P < 0.05 vs. 0 μmol/L, ^#^ *P* < 0.05 vs. 10 μmol/L A group, and ^Δ^ *P* < 0.05 vs. 30 μmol/L group). **(E)** Western blot analyses of the protein expression of Flag-Nrf2 in HK-2 cells after being transfected with or without empty plasmid or Nrf2 overexpression plasmid. **(F)** HK-2 cells were treated with 300 μmol/L PA for 24 h after being pretreated with tBHQ or transfected with Nrf2 overexpression plasmid and then stained with Mito SOX (red) and MitoTracker (green). **(G)** Quantification of the fluorescence intensities of MitoSOX in **(F)** (*n* = 3; *P < 0.05 vs. control group, ^#^ *P* < 0.05 vs. tBHQ group, ^Δ^ *P* < 0.05 vs. PA group, ^&^ *P*< 0.05 vs. PA + tBHQ group, and ^Ф^ *P*< 0.05 vs. empty vector group). **(H)** HK-2 cells were treated with 300 μmol/L PA for 24 h after being pretreated with tBHQ or transfected with Nrf2 overexpression plasmid and then stained with probe JC-1. **(I)** Quantification of the fluorescence intensities of JC-1 in **(H)** (*n* = 3; *P < 0.05 vs. control group, ^#^ *P* < 0.05 vs. tBHQ group, and ^Δ^ *P* < 0.05 vs. PA group). **(J)** HK-2 cells were treated with 300 μmol/L PA for 24 h after being pretreated with tBHQ or transfected with Nrf2 overexpression plasmid and then stained with MitoTracker (red) and an antibody against 8-OHdG.

We next investigated the effect of tBHQ, a well-characterized Nrf2 activator, on the Nrf2 signaling pathway in HK-2 cells. HK-2 cells were treated with tBHQ at different concentrations (0–50 μM), and as expected, tBHQ dose-dependently increased the protein level of Nrf2 (**Figures 5A–D**). Consistently, the protein levels of Nrf2 downstream targets, including HO-1 and NQQ-1, were increased in response to tBHQ (**Figures 5A–D**). Next, we incubated HK-2 cells with PA in the absence or presence of 30 μM tBHQ for 24 h and found that tBHQ ameliorated PA-induced mitochondrial ROS production (**Figures 5F, G**), mitochondrial injury (**Figures 5H, I**), and mitochondrial DNA oxidative damage (**Figure 5J**). Similarly, transfection of HK-2 cells with an Nrf2 overexpression plasmid (**Figure 5E**) significantly attenuated mitochondrial ROS production (**Figures 5F, G**) and reversed PA-induced mitochondrial membrane potential collapse (**Figures 5H, I**) and mitochondrial DNA oxidative damage (**Figure 5J**).

### 3.6 The Nrf2/ARE signaling pathway inhibited PA-induced mtROS-dependent NLRP3 inflammasome activation in HK-2 cells

**Figure 6:**
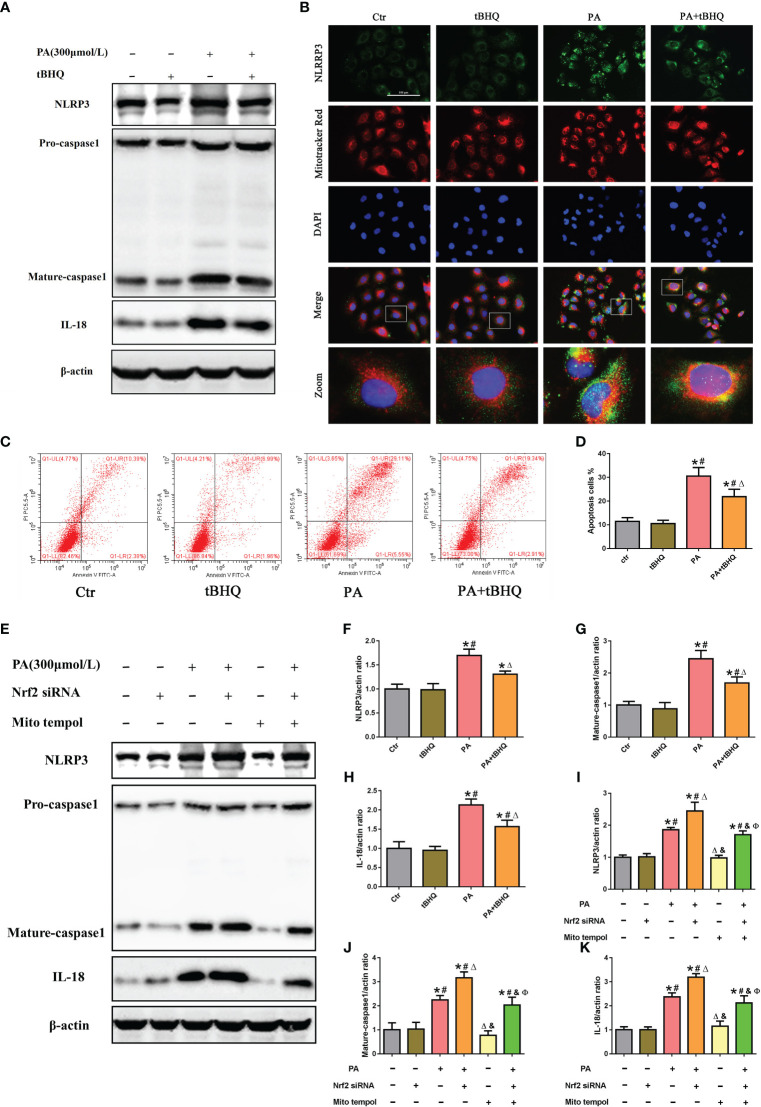
The Nrf2/ARE signaling pathway inhibited palmitic acid (PA)-induced mitochondrial ROS-dependent NLRP3 inflammasome activation in HK-2 cells. **(A)** Western blot analyses of the protein expression of NLRP3, caspase-1, and IL-18 in HK-2 cells after being pretreated with 30 μM tBHQ and then stimulated with 300 μmol/L PA for 24(h) **(B)** Representative images of immunofluorescence double-labeled NLRP3 and mitochondria (MitoTracker Red) in different groups. **(C)** Flow cytometry analysis of cell apoptosis in different groups. **(D)** Quantification analysis of apoptotic cells in **(C)** (*n* = 3; *P < 0.05 vs. control group, ^#^ *P* < 0.05 vs. tBHQ group, and ^Δ^ *P* < 0.05 vs. PA group). **(E)** Western blot analyses of the protein expression of NLRP3, caspase-1, and IL-18 in HK-2 cells after being pretreated with Mito Tempol or Nrf2 siRNA and then stimulated with 300 μmol/L PA for 24 (h) **(F–H)** Quantification analysis of NLRP3, caspase-1, and IL-18 expression in **(A)** (*n* = 3; *P < 0.05 vs. control group, ^#^ *P* < 0.05 vs. tBHQ group, and ^Δ^ *P* < 0.05 vs. PA group). **(I–K)** Quantification analysis of NLRP3, caspase-1, and IL-18 expression in **(E)** (*n* = 3; *P < 0.05 vs. control group, ^#^ *P* < 0.05 vs. Nrf2 siRNA group, ^Δ^ *P* < 0.05 vs. PA group, ^&^ *P*< 0.05 vs. PA + Nrf2 siRNA group, and ^Ф^ *P*< 0.05 vs. Mito Tempol group).

To further investigate the possible role of Nrf2 in NLRP3 inflammasome activation, PA-stimulated HK-2 cells were pretreated with tBHQ, as shown in **Figures 6A, F–H**. tBHQ treatment markedly inhibited the activation of the NLRP3 inflammasome in PA-treated HK-2 cells, as characterized by decreased NLRP3, caspase-1. and IL-18 levels. Moreover, we found that tBHQ significantly inhibited PA-induced colocalization of NLRP3 and MitoTracker (**Figure 6B**) and ameliorated PA-induced cell apoptosis in HK-2 cells (**Figures 6C, D**), demonstrating that the Nrf2/ARE signaling pathway plays a protective role in PA-induced cell apoptosis by inhibiting NLRP3 inflammasome activation in HK-2 cells. Next, we studied the underlying mechanism of the inhibitory effects of the Nrf2/ARE signaling pathway on NLRP3 inflammasome activation. Considering the regulatory effect of the Nrf2/ARE pathway on mtROS production, HK-2 cells were pretreated with Mito Tempol or/and Nrf2 siRNA, and knockdown of Nrf2 significantly increased the PA-induced expression of NLRP3, mature caspase-1, and IL-18 (**Figure 6E**). However, the enhancing effect of Nrf2 siRNA on NLRP3 inflammasome activation was markedly reversed by Mito Tempol intervention (**Figures 6E, I–K**). Collectively, these results indicated that Nrf2-induced NLRP3 inflammasome inhibition was dependent on mtROS production.

### 3.7 Activation of the Nrf2/ARE signaling pathway improved biochemical characteristics and morphological changes and ameliorated renal injury in HFD-induced obese rats

**Figure 7:**
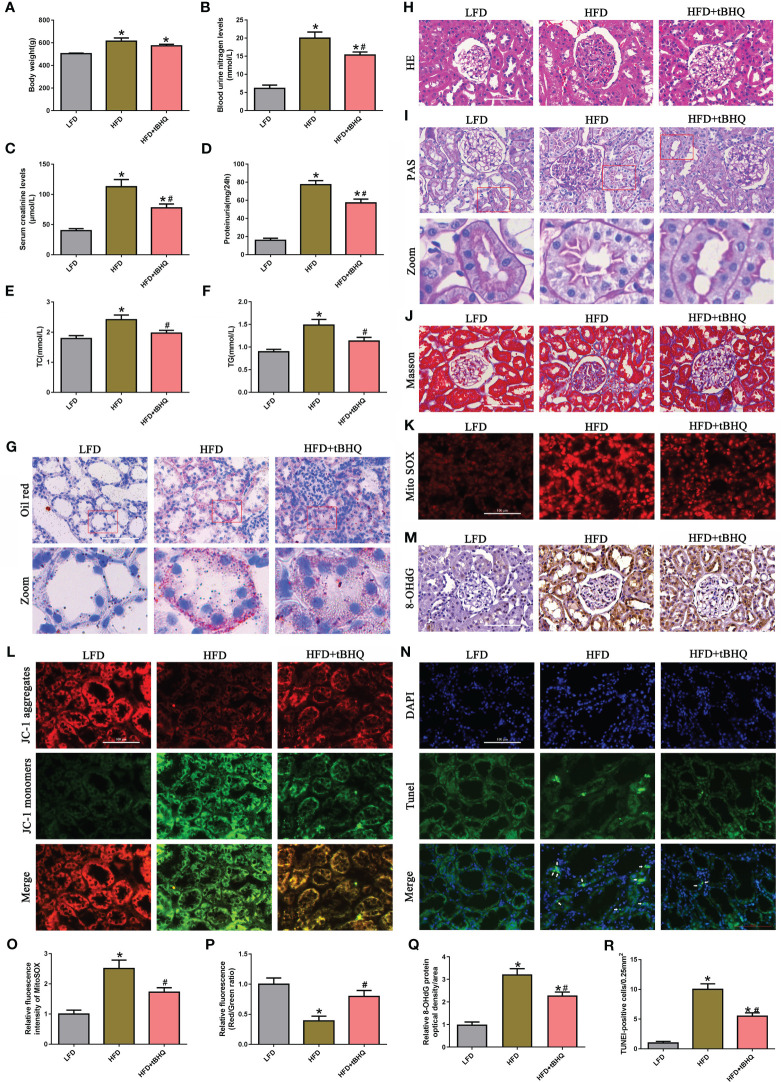
Activation of Nrf2/ARE signaling pathway improved the biochemical characteristics and morphological changes and ameliorated renal injury in high-fat diet (HFD)-induced obese rats. **(A–F)** Effects of tBHQ on body weight, BUN, SCr, urine protein, and TC and TG levels in HFD-induced obese rats (*n* = 6; *P < 0.05 vs. LFD group and ^#^ *P* < 0.05 vs. HFD group). **(G)** Representative images of Oil red O staining in kidney sections (×400). **(H–J)** Representative images of kidney sections subjected to H&E, PAS, and Masson staining. **(K)** Representative fluorescence images of kidney sections subjected to Mito SOX staining. **(L)** Representative fluorescence images of kidney sections subjected to JC-1 staining. **(M)** Representative image of immunohistochemical staining with 8-OHdG in kidney sections. **(N)** Kidney sections stained with TUNEL, apoptotic cells (arrowhead) are shown. **(O)** Quantification of the fluorescence intensities of Mito SOX in **(K)** (*n* = 6; *P < 0.05 vs. LFD group and ^#^ *P* < 0.05 vs. HFD group). **(P)** Quantification of the fluorescence intensities of JC-1 in **(L)** (*n* = 6; *P < 0.05 vs. LFD group and ^#^ *P* < 0.05 vs. HFD group). **(Q)** Quantification analysis of 8-OHdG expression in **(M)** (*n* = 6; *P < 0.05 vs. LFD group and ^#^ *P* < 0.05 vs. HFD group). **(R)** Quantification analysis of TUNEL-positive cells in **(N)** (*n* = 6; *P < 0.05 vs. LFD group and ^#^ *P* < 0.05 vs. HFD group).

As shown in **Figures 7A–F**, compared with those in LFD-fed rats, body weight, blood urea nitrogen (BUN), serum creatinine (SCr), urine protein, total cholesterol (TC), and triglyceride (TG) levels were significantly greater in HFD-fed rats; however, tBHQ intervention significantly attenuated these parameters, except for body weight, relative to those in HFD-fed rats. Oil red O staining showed that abnormal lipid accumulation was notably increased in renal proximal tubule cells of HFD-fed rats; however, tBHQ treatment markedly attenuated lipid deposition in renal proximal tubule cells in HFD-fed rats (**Figure 7G**). Additionally, H&E and PAS staining of the kidneys of HFD-fed rats revealed enhanced glomerular hypertrophy, mesangial proliferation, mesangial matrix deposition, and notable disruption of tubular epithelial cells compared to those of LFD-fed rats (**Figures 7H, I**). Masson trichrome staining revealed marked renal fibrosis in HFD-fed rats (**Figure 7J**); however, these changes were dramatically limited by tBHQ treatment. Moreover, mtROS generation (**Figures 7K, O**), ΔΨm loss (**Figures 7L, P**), and DNA oxidative damage (**Figures 7M, Q**) were enhanced in the kidneys of HFD-fed rats but markedly attenuated after tBHQ treatment. Furthermore, TUNEL staining demonstrated that the number of apoptotic cells (indicated by the arrow) was significantly reversed by tBHQ treatment (**Figures 7N, R**). Overall, these data demonstrated that tBHQ improved renal function in HFD-induced obese rats.

### 3.8 The Nrf2/ARE pathway inhibited NLRP3 inflammasome activation in the kidneys of HFD-induced obese rats

**Figure 8:**
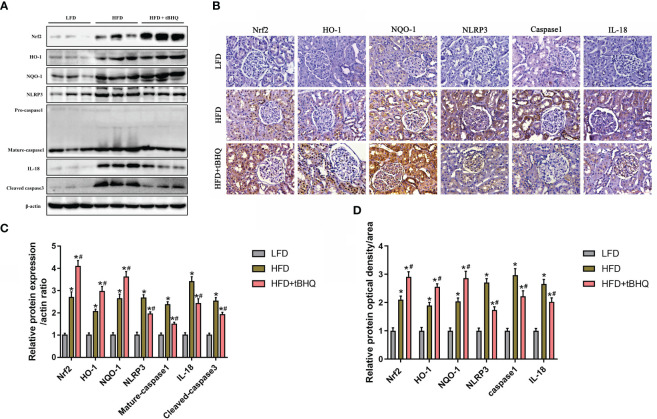
The Nrf2/ARE pathway inhibited NLRP3 inflammasome activation in the kidneys of high-fat diet (HFD)-induced obese rats. **(A)** Western blot analyses of the protein expression of Nrf2, HO-1, NQQ-1, NLRP3, caspase-1, and IL-18 in the kidneys of low-fat diet (LFD), HFD, and HFD rats treated with tBHQ. **(B)** Representative images of immunohistochemical staining for Nrf2, HO-1, NQQ-1, NLRP3, caspase-1, and IL-18 in kidney sections. **(C)** Quantification analysis of Nrf2, HO-1, NQQ-1, NLRP3, caspase-1, and IL-18 expression in **(A)** (*n* = 6; *P < 0.05 vs. LFD group and ^#^ *P* < 0.05 vs. HFD group). **(D)** Quantification analysis of Nrf2, HO-1, NQQ-1, NLRP3, caspase-1, and IL-18 expression in **(B)** (*n* = 6; *P < 0.05 vs. LFD group and ^#^ *P* < 0.05 vs. HFD group).

Finally, we investigated the role of the Nrf2/ARE pathway in the activation of the NLRP3 inflammasome in HFD-induced obese rats. Immunoblot analysis demonstrated that the expression of Nrf2, HO-1, NQO-1, NLRP3, mature-caspase-1, and IL-18 was significantly upregulated in the HFD group compared to the LFD group (**Figures 8A, C**). Additionally, the expression of Nrf2, HO-1, and NQQ-1 was significantly greater in HFD-fed rats after tBHQ intervention (**Figures 8A, C**). However, the HFD-induced increase in the expression of NLRP3, mature caspase-1, and IL-18 was significantly attenuated following treatment with tBHQ (**Figures 8A,C**). Subsequently, immunohistochemical staining (**Figures 8B, D**) also supported the above-mentioned results, indicating that the upregulation of the Nrf2/ARE pathway inhibited the activation of the NLRP3 inflammasome in the kidneys of HFD-induced obese rats.

## 4 Discussion

Yamagata et al. reported that the hazard ratio of proteinuria increased in CKD patients with hyperlipidemia of both genders, suggesting that serum lipid status abnormalities are considered independent risk factors for CKD progression (24). Previous findings have shown that ectopic lipid deposition in the kidney can cause structural and functional changes in proximal tubule cells, which contribute to the pathophysiology of kidney disease (25). Several underlying mechanisms have been linked to impaired autophagy flux, endoplasmic reticulum stress, oxidative stress, and inflammation (26, 27).

Numerous studies have demonstrated that the NLRP3 inflammasome is a key mechanism involved in the development of a wide variety of human kidney diseases, including AKI, CKD, glomerulonephritis, and obesity-related kidney disease (6, 28). Recently, research has revealed that high lipid levels activate the CD36–NLRP3 inflammasome axis and promote the release of the inflammatory cytokines IL-1β and IL-18, which induce FFA-related renal tubular injury (9). In the present study, we also found that high lipid levels activated the NLRP3 inflammasome both *in vivo* and *in vitro*, which induced tubular epithelial cell apoptosis. However, the exact mechanism of NLRP3 inflammasome activation in hyperlipidemia-induced kidney injury has not yet been defined. Previous research has revealed that the NLRP3 inflammasome can be activated by various cellular signals, including reactive oxygen species (ROS), oxidized DNA, potassium efflux, calcium influx, and lysosomal damage (29, 30). Emerging evidence indicates that mitochondrial dysfunction plays a major role in the activation of the NLRP3 inflammasome. Furthermore, the production of ROS from damaged mitochondria was proposed to promote the deubiquitylation of NLRP3 and subsequently contribute to NLRP3 inflammasome activation (31). Moreover, previous research has shown that mitochondria can function as a platform for assembling NLRP3 inflammasome components to enable the activation of caspase-1 (22, 23). In this study, we observed that the NLRP3 inflammasome was activated both *in vivo* and *in vitro* and was accompanied by increased translocation of NLRP3 to the mitochondria in HK-2 cells treated with PA. Moreover, the mitochondrion-targeted antioxidant Mito Tempol decreased the activation of the NLRP3 inflammasome and the recruitment of NLRP3 within mitochondria by reducing mitochondrial ROS generation and DNA oxidative damage. These results indicated that mitochondrial ROS and oxidized DNA are the signals for triggering the NLRP3 inflammasome in high-lipid-induced renal tubular injury. Thus, targeting the mechanism preserving the mitochondrial redox balance and thereby inhibiting NLRP3 inflammasome activation in CKD patients with hyperlipidemia may be an attractive therapeutic approach.

The Nrf2/ARE pathway is considered a pivotal antioxidant defense system that plays a key role in regulating the status of cellular oxidative stress. Numerous studies have described the association of the Nrf2/ARE pathway with kidney diseases. Wang et al. showed that activation of the Nrf2 signaling pathway ameliorates sepsis-induced AKI (32). Moreover, Jiang et al. suggested that Nrf2 plays a crucial role in ameliorating streptozotocin-induced renal damage in DKD mice and that genetic deletion of Nrf2 significantly increased ROS generation and oxidative DNA damage and accelerated renal injury in STZ-induced DKD mice (33). In the present study, we demonstrated that the Nrf2/ARE signaling pathway is activated in HK-2 cells under high lipid conditions and that the expression of Nrf2 was also notably upregulated in the kidneys of CKD patients with hyperlipidemia. In addition, Nrf2 silencing exacerbated PA-induced mtROS production and mitochondrial damage; however, Nrf2 activation with tBHQ or Nrf2 overexpression plasmids significantly alleviated cell injury by reducing mtROS production, improving mitochondrial membrane potential, and ameliorating oxidative DNA damage in HK-2 cells, and these effects were further verified in the kidneys of HFD-induced obese rats after tBHQ intervention.

The Nrf2/ARE pathway is a pivotal antioxidant defense system, and its effect on hyperlipidemia-induced renal inflammation and the potential underlying mechanism remain unclear. Recently, numerous studies have focused on Nrf2 signaling and the NLRP3 inflammasome. It has been demonstrated that activation of Nrf2 signaling negatively regulates NLRP3 inflammasome activity in diabetic retinopathy (34) and cerebral ischemia−reperfusion injury (35). In kidney research, Szeto et al. demonstrated that the stabilization of endogenous Nrf2 by minocycline attenuated NLRP3 inflammasome activation in diabetic nephropathy (36). Mahmoud et al. showed that Nrf2/ARE/HO-1 signaling suppressed the NF-κB/NLRP3 inflammasome axis and improved kidney function in methotrexate (MTX)-induced nephrotoxicity (37). Here we demonstrated that Nrf2/ARE antioxidant signaling plays a key role in the activation of the NLRP3 inflammasome in hyperlipidemia-induced renal injury. When HK-2 cells were exposed to high-lipid media, Nrf2 activation induced by tBHQ markedly mitigated PA-induced NLRP3 inflammasome activation; in contrast, Nrf2 knockdown significantly enhanced PA-induced NLRP3 inflammasome activation in HK-2 cells, suggesting that Nrf2/ARE signaling plays a potential role in negatively regulating NLRP3 inflammasome activation in high-lipid-induced HK-2 cells. Nonetheless, the underlying mechanism by which Nrf2/ARE signaling negatively regulates NLRP3 inflammasome activation remains unclear. Therefore, we further found that the enhancing effect of Nrf2 siRNA on NLRP3 inflammasome activation was markedly reversed by Mito Tempol in HK-2 cells, demonstrating that the potential mechanism of Nrf2-induced NLRP3 inflammasome inhibition was dependent on reducing mtROS production. However, the influence of the Nrf2/ARE pathway on other inflammatory signaling pathways, such as the NF-κB pathway, JAK-STAT pathway, and MAPK pathway, should be clarified in a future work.

tBHQ, a well-characterized Nrf2 activator, can maintain the stability of the Nrf2 protein through inhibition of Keap1-mediated ubiquitination. Previous studies have revealed that tBHQ plays a protective role against hypoxic–ischemic brain damage (38), cisplatin-induced nephrotoxicity (39), and hepatic ischemia/reperfusion (I/R) injury (40) by activating Nrf2-mediated antioxidative signaling pathways. In our study, we also found that tBHQ attenuated hyperlipidemia-induced renal injury, and the potential protective mechanism of tBHQ involved inhibiting mtROS–NLRP3 inflammasome activation *via* the upregulation of Nrf2/ARE-related antioxidant signaling. Accordingly, we proposed that the Nrf2/ARE antioxidant signaling–mitochondrial ROS–NLRP3 inflammasome pathway is involved in hyperlipidemia-induced renal injury. Furthermore, other Nrf2 agonists, such as bardoxolone methyl, are being evaluated in ongoing clinical trials for kidney disease, including DKD (41), Alport syndrome (AS), and autosomal dominant polycystic kidney disease (ADPKD) (42), providing great therapeutic potential and expectations for the future of Nrf2 agonist development for kidney diseases.

Finally, we showed that the Nrf2/ARE signaling pathway was activated under high lipid conditions both *in vitro* and *in vivo*. Therefore, why did the activation of the Nrf2 antioxidant response fail to protect against tubular cell damage under high lipid conditions? We believe that activation of the Nrf2 antioxidant response plays a key role in preventing mtROS production, preserving mitochondrial function and inhibiting NLRP3 inflammasome activation during the early stage of high-lipid-induced tubular cell damage. However, at the late stage of high-lipid-induced cell damage, the Nrf2-mediated protective mechanism is saturated by persistent and excessive ROS, resulting in NLRP3 inflammasome activation and tubular cell and renal damage.

## 5 Conclusion

**Figure 9:**
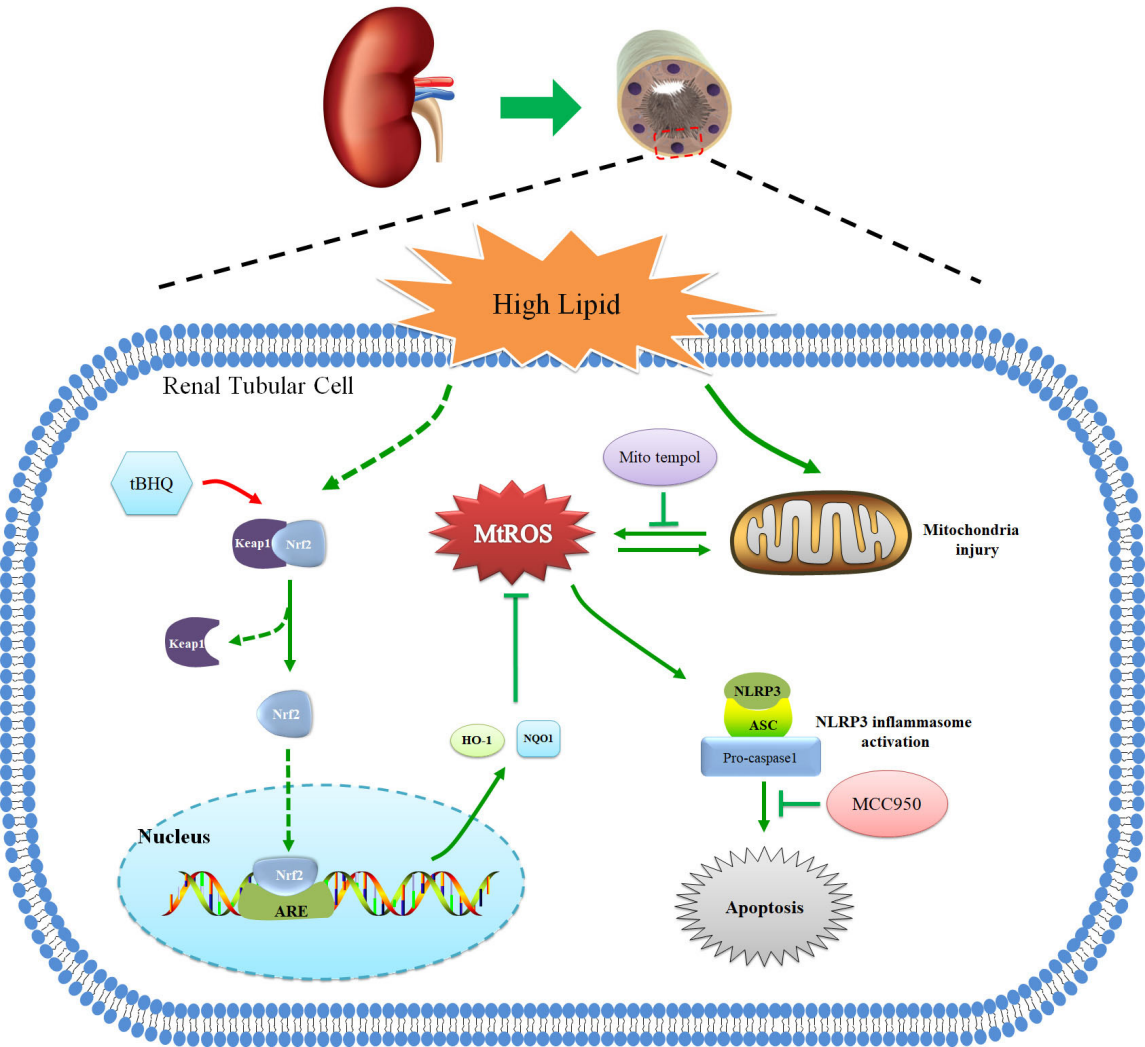
Schematic diagram of Nrf2/ARE signaling pathway, mitochondrial ROS, and NLRP3 inflammasome in renal tubular epithelial cells under high lipid conditions. Activation of Nrf2/ARE antioxidant signaling protects against high-lipid-induced renal tubular epithelial cell damage by inhibiting mtROS-mediated NLRP3 inflammasome activation.

In summary, we demonstrated that hyperlipidemia induced Nrf2/ARE signaling activation, mitochondrial ROS production, and NLRP3 inflammasome activation. Activation of the mtROS–NLRP3 inflammasome pathway is a critical contributor to renal tubular epithelial cell injury in CKD patients with hyperlipidemia. Activation of Nrf2/ARE antioxidant signaling protects against high-lipid-induced renal tubular epithelial cell damage by inhibiting mtROS-mediated NLRP3 inflammasome activation (**Figure 9**). Thus, our findings highlight the potential for the pharmacological activation of the Nrf2/ARE antioxidant pathway, such as through the use of tBHQ as a therapeutic approach against oxidative stress and inflammation in CKD patients with hyperlipidemia.

## Data availability statement

The original contributions presented in the study are included in the article/supplementary material. Further inquiries can be directed to the corresponding authors.

## Ethics statement

The studies involving humans were approved by The Ethics Committee of The First Affiliated Hospital of Chongqing Medical University. The studies were conducted in accordance with the local legislation and institutional requirements. The participants provided their written informed consent to participate in this study. The animal study was approved by The Ethics Committee of The First Affiliated Hospital of Chongqing Medical University. The study was conducted in accordance with the local legislation and institutional requirements.

## Author contributions

X-sJ: Funding acquisition, Data curation, Formal Analysis, Investigation, Writing – original draft. TL: Investigation, Methodology, Software, Writing – review & editing. Y-fX: Software, Data curation, Formal Analysis, Resources, Writing – review & editing. HG: Data curation, Formal Analysis, Resources, Software, Writing – review & editing. WR: Conceptualization, Supervision, Writing – review & editing. X-gD: Conceptualization, Supervision, Funding acquisition, Writing – review & editing.
